# Supplementary material for: The Rad51 paralogs facilitate a novel DNA strand specific damage tolerance pathway
Source: Nat Commun. 2019 Aug 5;10:3515. doi: 10.1038/s41467-019-11374-8 (PMC6683157; doi:10.1038/s41467-019-11374-8)
Supplement: Supplementary file 1 — Supplementary Information [file 41467_2019_11374_MOESM1_ESM.pdf]

Supplementary Information

The Rad51 paralogs facilitate a novel DNA strand specific damage tolerance pathway

Rosenbaum J.C.[1,*], Bonilla B.[2,*], Hengel S.R. [2,*], et al.

Supplementary Figures, Supplementary Tables, Supplementary References

Supplementary Figure 1

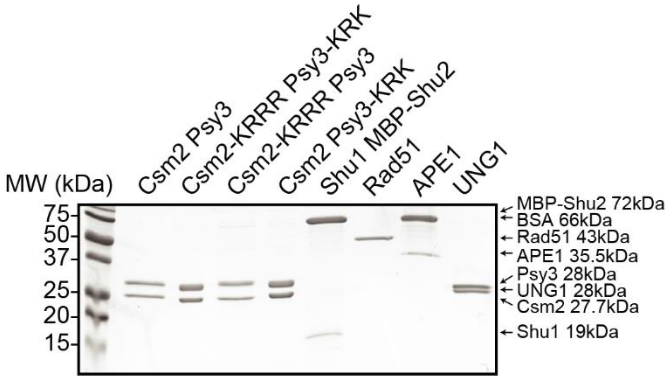

Supplementary Figure 1: Gel of recombinant proteins. An SDS-PAGE gel (15%) showing purified protein complexes of Csm2-Psy3 (27.7 kDa, 28kDa), Csm2-KRRR Psy3-KRK, Csm2-KRRR Psy3, Csm2 Psy3-KRK, Shu1-MBP-Shu2 (19 kDa, 72 kDa), Rad51 (43 kDa), APE1 and BSA (35.5 kDa, 66 kDa), and UNG1 (28 kDa) protein. All protein complexes were purified to near homogeneity except APE1 and UNG1, which were purchased from NEB and Abcam, respectively. Contrast is enhanced equally in the entire image and unprocessed images are provided.

Supplementary Figure 2

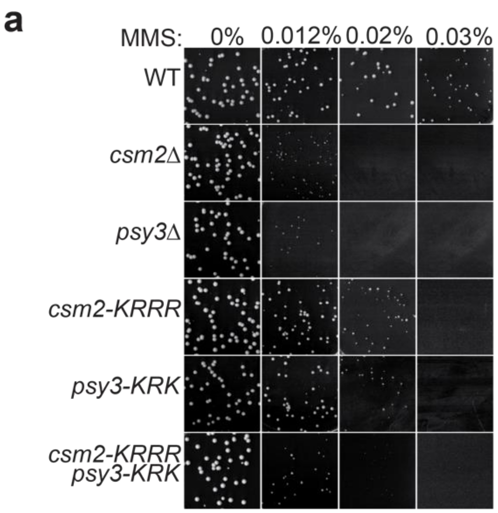

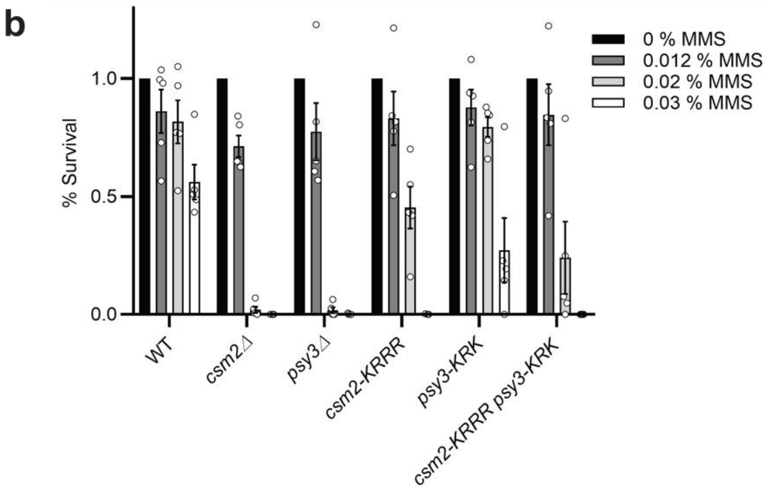

Supplementary Figure 2: Viability of Csm2 and Psy3 DNA binding mutants upon MMS exposure. (**a**) Representative images of the indicated genotypes used for serial dilution viability assays. All strains were exposed to MMS for 2 days at 30˚C. (**b**) Viability as determined by cell counts on three plates for Csm2 and Psy3 DNA binding mutants per condition. The experiment was repeated five times with standard error of the mean plotted.

Supplementary Figure 3

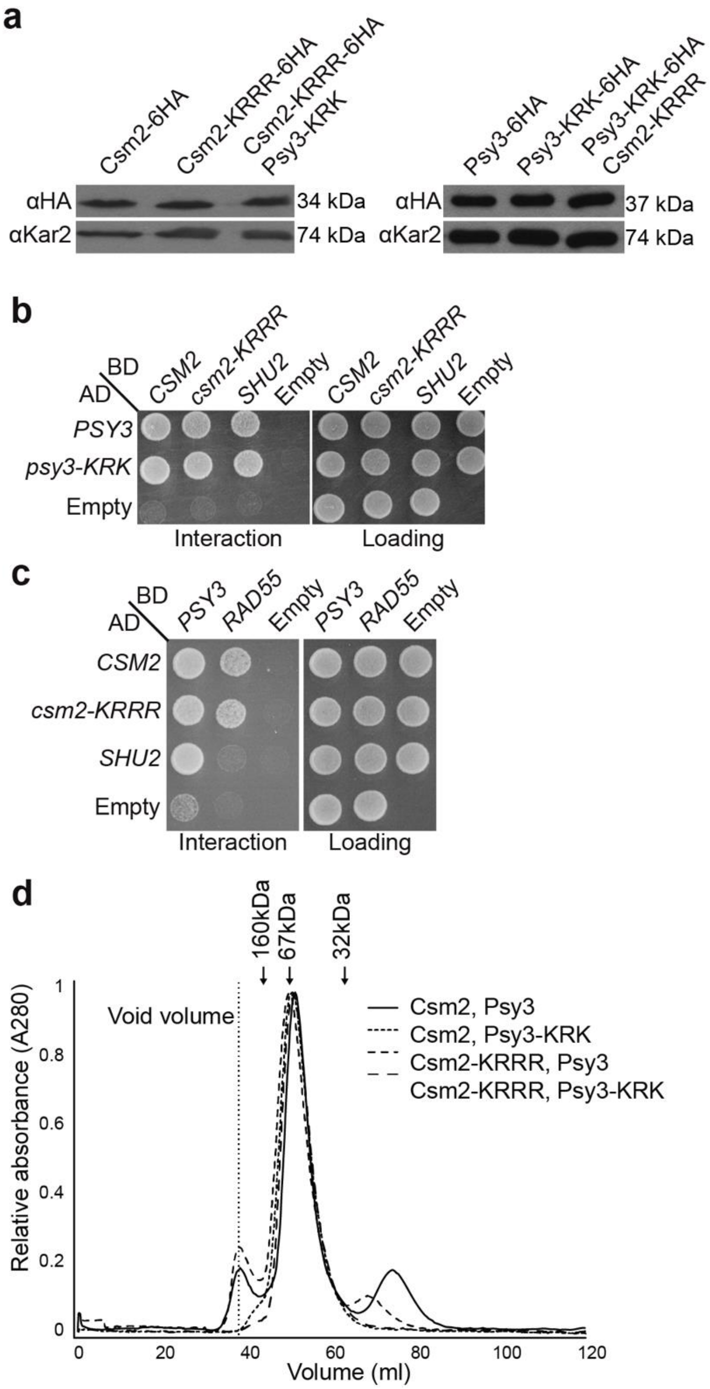

Supplementary Figure 3: Csm2-Psy3 DNA binding mutants express normally and retain their protein-protein interactions. (**a**) Mutating the DNA binding residues in Csm2 and Psy3 does not affect protein expression. Protein was extracted from equal

cell numbers and protein expression levels of the indicated 6HA tagged strains were determined by protein blot using an HA antibody. Expression of Kar2 was used as a loading control. (**b**) Csm2 and Psy3 DNA binding mutants exhibit wild-type yeast-2-hybrid (Y2H) interactions. Y2H analysis of pGAD-*PSY3*, pGAD-*psy3-KRK*, or pGAD-C1 (Empty) with pGBD-*CSM2*, *csm2-KRRR, SHU2,* or pGBD-C1 (Empty). A Y2H interaction is indicated by plating equal cell numbers on SC medium lacking histidine, tryptophan, and leucine. Equal cell loading is determined by plating on SC medium lacking tryptophan and leucine used to select for the pGAD (AD) and pGBD (BD) plasmids. (**c**) Y2H interactions performed as in (**b**), with analysis of pGAD-*CSM2*, pGAD-*csm2-KRRR*, pGAD-*SHU2*, or pGAD-C1 (Empty) with pGBD-*PSY3*, pGBD-*RAD55*, or pGBD-C1 (Empty). (**d**) Csm2 and Psy3 DNA binding mutants form a stable heterodimer with similar properties as wild-type. Overlaid chromatograms show the elution profile of Csm2-Psy3 heterodimers for wild-type and DNA binding mutant combinations resolved using size exclusion chromatography.

Supplementary Figure 4

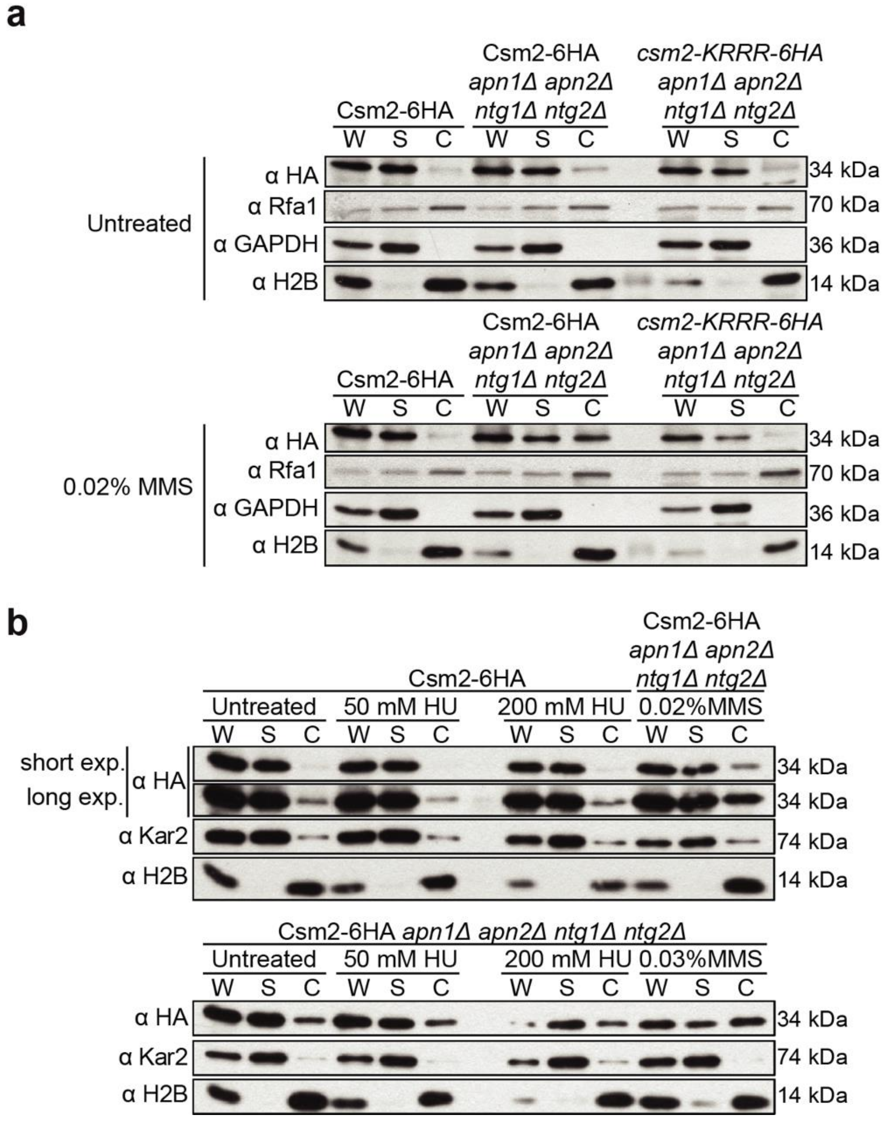

Supplementary Figure 4: Csm2 chromatin enrichment is MMS specific. (**a**) Unlike Csm2, RPA chromatin association is independent of Csm2 DNA binding. Csm2-6HA expressing cells were synchronized in G1 with alpha-factor and released into YPD medium or YPD medium containing 0.02% MMS for 1 hour before cellular fractionation. Csm2 protein levels from whole cell extract (W), supernatant (S) and chromatin (C) fractions from the indicated strains were determined by western blot using HA antibody.

RPA chromatin association was assessed using a Rfa1-specific antibody. GAPDH and histone H2B were used as fractionation controls (S and C, respectively). (**b**) Csm2 is not chromatin enriched upon non-template induced replication stress. Csm2-6HA or Csm2-6HA *apn1*Δ *apn2*Δ *ntg1*Δ *ntg2*Δ cells were synchronized in G1 with alpha-factor and released into YPD medium or YPD medium containing MMS (0.02% or 0.03%) or HU (50 mM or 200 mM) for 1 hour before cellular fractionation. Csm2 protein levels from whole cell extract (W), supernatant (S) and chromatin (C) fractions from the indicated strains were determined by western blot using HA antibody. Kar2 and histone H2B were used as fractionation controls (S and C, respectively).

Supplementary Figure 5

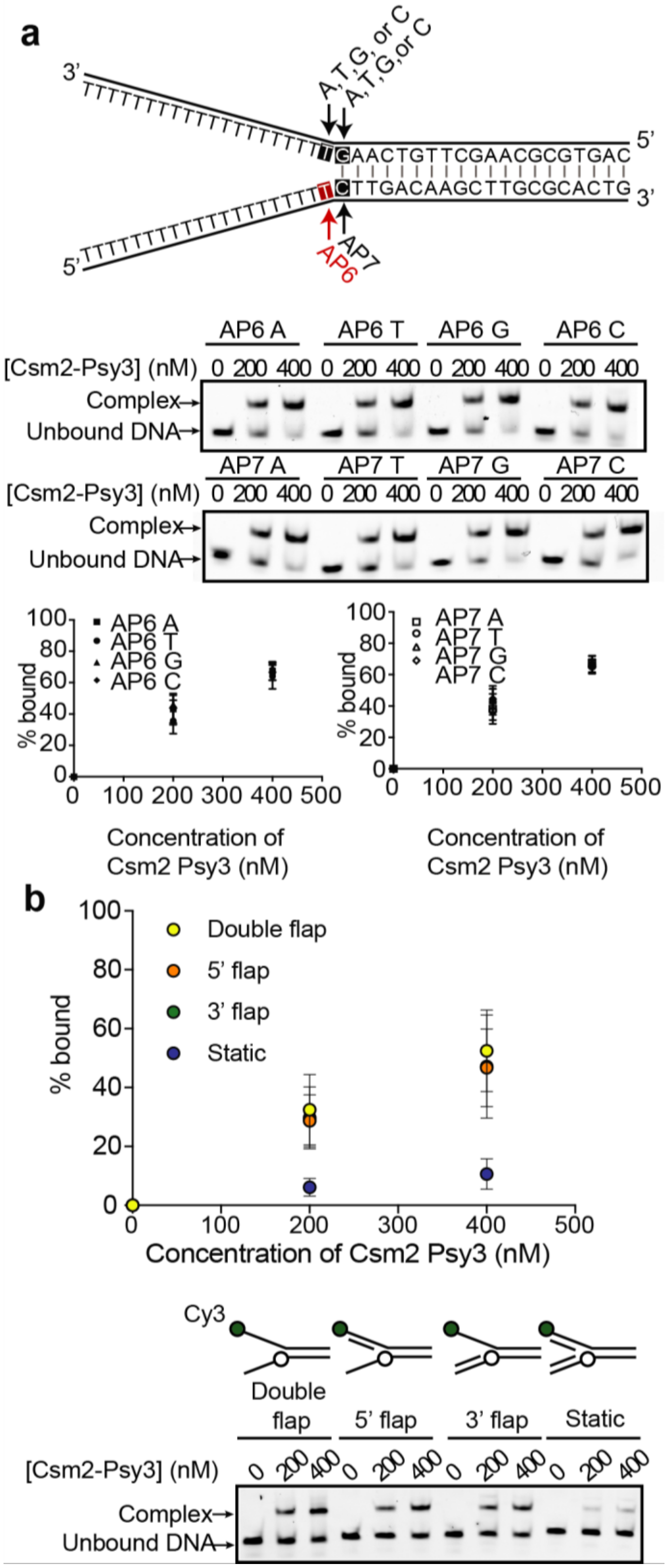

Supplementary Figure 5: Binding reactions of Csm2-Psy3 for varying AP6 and AP7 substrates. (**a**) Csm2-Psy3 binds to double-flap substrates with abasic site analogs independently of DNA sequence opposite the abasic site analog. DNA binding by Csm2-Psy3 was measured by EMSA, with increasing concentrations of the Csm2-Psy3 heterodimer added to 5 nM Cy3-labelled double-flap substrates containing A, T, G, or C nucleotide opposite the abasic site analog in AP6 or AP7. The percent of Csm2-Psy3 bound to the substrate was quantitated and compared to total intensity in each lane. Error bars indicate standard deviations measured in quadruplicate experiments. (**b**) Binding reactions were performed with increasing concentrations of Csm2-Psy3 using 25 nM of the indicated substrates [AP6 containing double-flap (yellow), 5' flap (orange), 3' flap (green), and static replication fork (blue)][1] and run on a polyacrylamide gel. Quantification of Csm2-Psy3 bound to the different flap substrates was performed by dividing the % bound complex by the total substrate intensity in each lane. Error bars represent standard deviations and these experiment was performed four times. Contrast is enhanced equally in the entire image and unprocessed images are provided.

Supplementary Figure 6

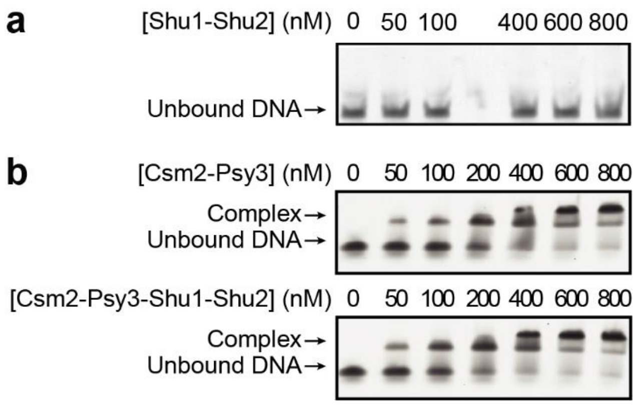

Supplementary Figure 6: Shu1-Shu2 does not bind AP6. (**a**) Binding reactions were performed with 25 nM AP6 and increasing concentrations of Shu1-Shu2 (50, 100, 400, 600, and 800 nM) and analysed by EMSA. (**b**) Same as (**a**) except that Csm2-Psy3 or Csm2-Psy3-Shu1-Shu2 were analysed. Contrast is enhanced in image equally across entire image and unprocessed image is provided. Note that reactions were run on acrylamide gels containing 5% glycerol which can change the mobility of the complex. Contrast is enhanced equally in the entire image and unprocessed images are provided.

Supplementary Figure 7

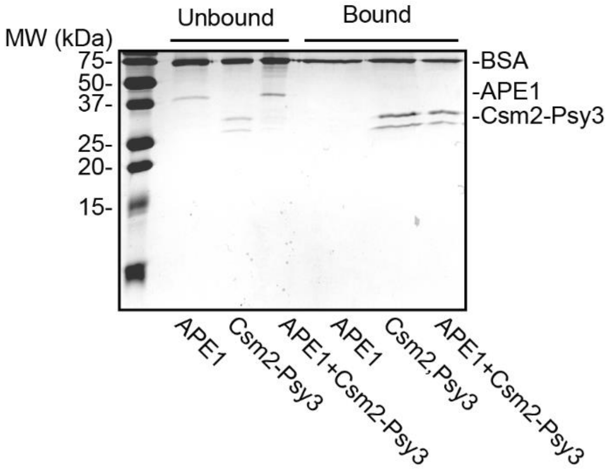

Supplementary Figure 7: Csm2-Psy3 does not bind APE1 enzyme by *in vitro* pull-down. HIS-tagged Csm2-Psy3 (1 µM) was incubated with APE1 (1 µM) and Csm2-Psy3 was pulled down using Ni-NTA beads. The unbound and bound proteins were analyzed by SDS-PAGE analysis. APE1 and Csm2-Psy3 alone were used as controls. BSA was included in the binding reaction buffer. Contrast is enhanced equally in the entire image and unprocessed images are provided.

Supplementary Figure 8

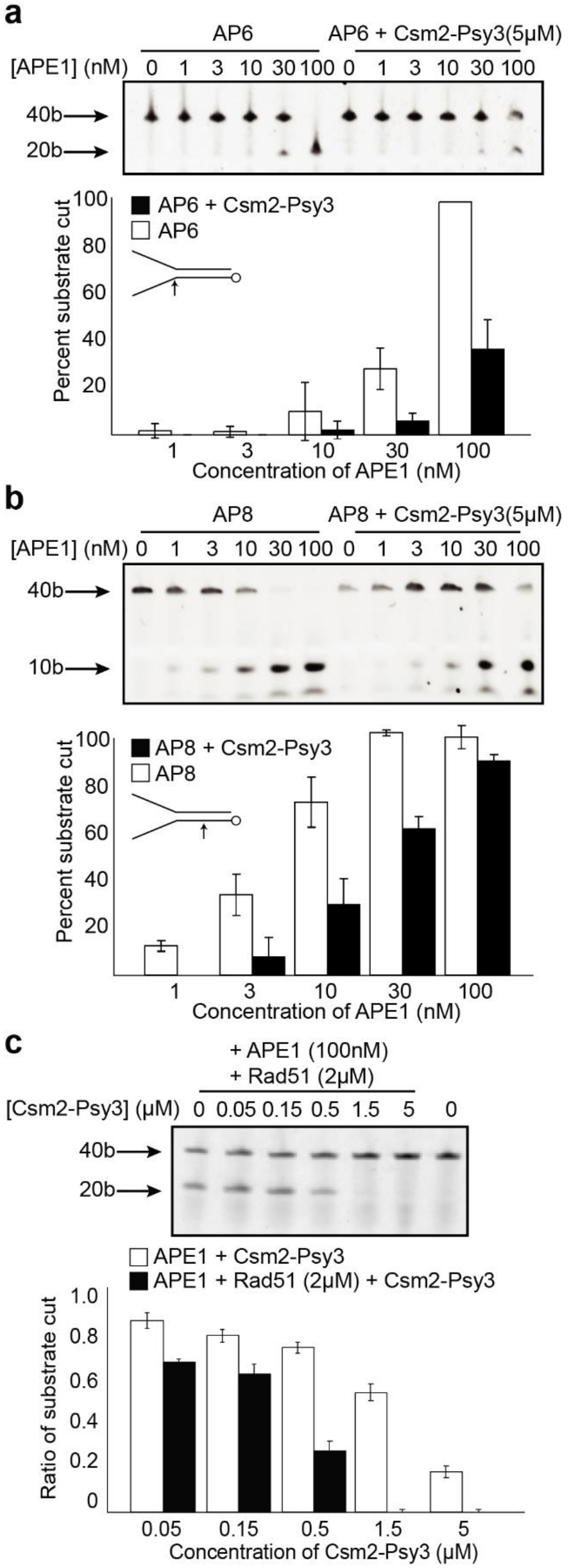

Supplementary Figure 8: Csm2-Psy3 protect double-flap substrates proximal abasic site analogs from APE1 cleavage. (**a**) Representative gel (top) and chart representing triplicate experiments (bottom) showing that APE1 (100 nM) activity against the double-flap substrates containing an abasic site analog on the 3' oligo at the junction (AP6) is inhibited by the presence of Csm2-Psy3 (5 µM). Error bars in the chart indicate the 95% confidence interval across all experiments. (**b**) Results from the assay described in (a) using substrate AP8, which contains an abasic site analog located in the duplex region 10 bases (approximately 34 Å) from the junction. APE1 is considerably more active against the double-stranded DNA substrate but is still inhibited by Csm2-Psy3. (**c**) Both Csm2-Psy3 and Rad51 protect AP6 against cleavage by APE1. Representative gel and bar graph showing increased protection of substrate AP6 (100 nM) from APE1 endonuclease activity (100 nM) by Csm2-Psy3 (0 to 5 µM) when Rad51 (2 µM) is also present. Error bars in the graph represent standard deviations from three experiments. Contrast is enhanced equally in the entire image and unprocessed images are provided.

Supplementary Figure 9

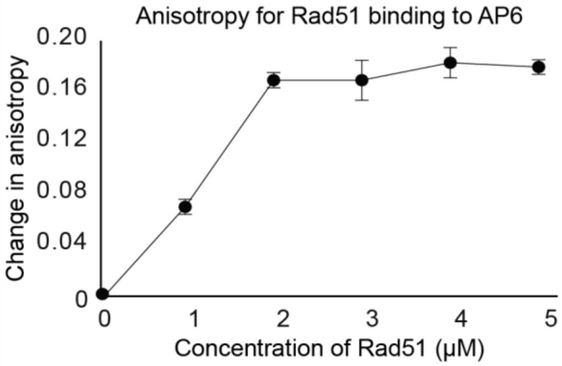

Supplementary Figure 9: Rad51 binds AP6 substrate in cleavage assay buffer. Anisotropy binding assays were performed with AP6 (20 nM) substrate titrated with increasing concentrations yeast Rad51 protein in 20 mM Tris (pH 8.0), 50 mM NaCl, 5 mM $MgOAc_2$, 5% glycerol, 1 mM DTT, 2 mM ATP, and 0.5 mg per mL BSA to a final volume of 100 μL. Since Rad51 binds both ssDNA and dsDNA, we observe saturation of these binding events at 2 μM.

Supplementary Table 1: Dissociation constants for Csm2-Psy3 DNA binding mutants.

Name	Dissociation constant (K_d)	B_{max}
WT (3' strand)	435 +/- 37	0.28 +/- 0.01
Csm2 Psy3-KRK	2828 +/- 512	0.4 +/- 0.0
Csm2-KRRR Psy3	N.D.	N.D.
Csm2-KRRR Psy3	N.D.	N.D.

Supplementary Table 2: Dissociation constants for DNA substrates containing abasic sites analogs.

**Name**	**Dissociation constant (K_d)**	**B_{max}**
WT (3' strand)	127 +/- 10	0.28 +/- 0.01
AP1	105 +/- 9	0.27 +/- 0.01
AP2	127 +/- 12	0.28 +/- 0.01
AP3	104 +/- 10	0.27 +/- 0.01
AP4	131 +/- 18	0.28 +/- 0.01
WT (5'strand)	122 +/- 5	0.31 +/- 0.03
AP5	142 +/- 12	0.31 +/- 0.01
AP6	72 +/- 7	0.34 +/- 0.01
AP7	112 +/- 13	0.32 +/- 0.01
AP8	153 +/- 16	0.33 +/- 0.01

Supplementary Table 3: Yeast strains and plasmids.

Yeast strains				
**Simplified genotype used in figures and text**	**Strain ID**	**Genotype**	**Strain Background**	**Reference**
WT (wild-type)	W9100-17D	*MAT***a** *ADE2 leu2-3,112 his3-11,15 ura3-1 TRP1 lys2Δ RAD5*	W303	[2]
csm2Δ	KBY107-2D	*MAT***a** *csm2Δ::KanMX LYS2*	W303	[2]
psy3Δ	KBY108-3D	*MAT***a** *psy3Δ::KanMX4 trp1-1 LYS2*	W303	[3]
csm2-KRRR	KBY820-1	*MAT***a** *csm2-K189A,R190A,R191A,R192A trp1-1 LYS2*	W303	This study
psy3-KRK	KBY909-1	*MATα psy3-K199A,R200A,R201A trp1-1 LYS2*	W303	This study
csm2-KRRR psy3-KRK	KBY945-3A	*MAT***a** *psy3-K199A,R200A,K201A csm2-K189A,R190A,R191A,R192A LYS2 trp1-1*	W303	This study
csm2-6xHA	KBY530-1	*MATα CSM2-6HA-k.i.TRP1 LYS2 trp1-1*	W303	This study
csm2-KRRR-6xHA	KBY1106-1	*MAT***a** *csm2-K189A,R190A,R191A,R192A-6HA-k.i.TRP1 LYS2 trp1-1*	W303	This study
csm2-KRRR-6xHA psy3-KRK	KBY1108-1	*MAT***a** *psy3-K199A,R200A,K201A csm2-K189A,R190A,R191A,R192A-6HA-k.i.TRP1 LYS2 trp1-1*	W303	This study

psy3-6xHA	KBY565-1A	*MAT**a** PSY3-6HA-k.i.TRP1 trp1-1*	W303	This study
psy3-KRK-6xHA	KBY1105-1	*MAT**a** psy3-K199A,R200A,K201A-6HA-k.i.TRP1*	W303	This study
psy3-KRK-6xHA csm2-KRRR	KBY1107-1	*MAT**a** psy3-K199A,R200A,K201A-6HA-k.i.TRP1 csm2-K189A,R190A,R191A,R192A LYS2 trp1-1*	W303	This study
	PJ69-4A*	*MAT**a** trp1-901 leu2-3,112 ura3-52 his3-200 gal4∆ gal80∆ GAL2-ADE2 LYS2::GAL1-HIS3 met2::GAL7-lacZ*		[4]
	PJ69-4α*	*MATα trp1-901 leu2-3,112 ura3-52 his3-200 gal4∆ gal80∆ GAL2-ADE2 LYS2::GAL1-HIS3 met2::GAL7-lacZ*		[4]
WT	ySR_128	*MATα lys2Δ(Chr.II) ura3Δ(Chr.V) can1Δ(Chr.V) ade2Δ(Chr.XV) leu2-3,112 trp1-289 his7-2 Chr.II 488694::lys2::ADE2-URA3-CAN1*	CG379	[5]
ung1Δ	ySR_616	*MATα ung1::NatMX*	CG379	[6]
csm2Δ	yTM_62	*MATα csm2::HygMX*	CG379	This study
psy3Δ	yTM_54	*MATα psy3::HygMX*	CG379	This study
ung1Δ csm2Δ	yTM_70	*MATα ung1::NatMX csm2::HygMX*	CG379	This study
ung1Δ psy3Δ	yTM_52	*MATα ung1::NatMX psy3::HygMX*	CG379	This study

WT	KBY869-1C	*MAT***a** *CAN1 trp1-1 LYS2*	W303	[3]
csm2Δ	KBY869-8A	*MATα csm2::KanMX4 CAN1 trp1-1 LYS2*	W303	[3]
psy3Δ	KBY758-11A	*MAT***a** *psy3::KanMX4 CAN1 LYS2 trp1-1*	W303	[3]
csm2-KRRR	KBY1080-1B	*MAT***a** *psm2-K189A,R190A,R191A,R192A LYS2 CAN1*	W303	This study
psy3-KRK	KBY1081-4B	*MATα psy3-K199A,R200A,K201A CAN1*	W303	This study
csm2-KRRR psy3-KRK	KBY1101-2D	*MAT***a** *psy3-K199A,R200A,K201A csm2-K189A,R190A,R191A,R192A CAN1*	W303	This study
csm2-6xHA	KBY809-4C	*MAT* **a** *ADE2 LYS2 Csm2-6HA-k.l.TRP1*	W303	[3]
csm2-6xHA apn1Δ apn2Δ ntg1Δ ntg2Δ	KBY1179-17D	*MAT***a** *ADE2 trp1-1 LYS2 Csm2-6HA-k.l.TRP1 ntg1::NatMX ntg2::NatMX apn1::HygMX apn2::HygMX*	W303	This study
csm2-KRRR-6xHA apn1Δ apn2Δ ntg1Δ ntg2Δ	KBY1219-15D	*MAT* **a** *ADE2 TRP1 csm2-K189A,R190A,R191A,R192A-6HA-k.i.TRP1 LYS2 ntg1::NatMX ntg2::NatMX apn1::HygMX apn2::HygMX*	W303	This study
apn1Δ apn2Δ ntg1Δ ntg2Δ	KBY698-4A	*MATα ntg1::NatMX, ntg2::NatMX ,apn1::HygMX apn2::HygMX LYS2*	W303	[3]

apn1Δ apn2Δ ntg1Δ ntg2Δ csm2Δ	KBY745-17C	*MAT***a** *csm2::KanMX ntg1::NatMX ntg2::NatMX apn1::HygMX apn2::HygMX LYS2*	W303	This study
apn1∆ apn2∆ ntg1∆ ntg2∆ csm2-KRRR psy3-KRK-6HA	KBY1187-41D	*MATα psy3-K199A,R200A,K201A-6HA-k.i.TRP1 csm2-K189A,R190A,R191A,R192A ntg1::NatMX ntg2::Nat apn1::HygMX apn2::HygMX LYS2*	W303	This study
**Plasmids**				
**Name**	**Purpose**	**Backbone**	**Selection marker**	
pAG32	HygMX knock out cassette	pFA6	Ampicillin/HYG	[7]
pTM-19	empty vactor/ APOBEC3B mutagenesis	pySR-419	Ampicillin/LEU2	This study
pTM-21	APOBEC3B expression/ APOBEC3B mutagenesis	pSR-440	Ampicillin/LEU2	This study
pGAD-csm2-K189A,R190A,R191A,R192A	Y2H	pGAD-C1	Ampicillin/LEU2	This study
pGBD-csm2-K189A,R190A,R191A,R192A	Y2H	pGBD-C1	Ampicillin	This study
pGAD-PSY3-K199A-R200A-K201A	Y2H	pGAD-C1	Ampicillin/LEU2	This study
pGBD-psy3-K199A,R200A,K201A	Y2H	pGBD-C1	Ampicillin	This study
pGAD-SHU2	Y2H	pGAD	Ampicillin/LEU2	[8]
pGBK-SHU2	Y2H	pGBK	Kanamicin	[2]

pGAD-CSM2	Y2H	pGAD	Ampicillin/LEU2	[8]
pGBK-CSM2	Y2H	pGBK	Kanamicin	[8]
pGAD-PSY3	Y2H	pGAD	Ampicillin/LEU2	[8]
pGBK-PSY3	Y2H	pGBK	Kanamicin	[8]
pGBD-RAD55	Y2H	pGBD	Ampicillin	[8]
pGAD-C2	Y2H empty vector	N/A	Ampicillin/LEU2	
pGBD-C1	Y2H empty vector	N/A	Ampicillin	
pYM3	6xHA cassette	pYM	Ampicillin/TRP1	[9]
YIplac211-csm2-K189A,K190A,R191A,R192A	Yeast integration	YIplac211	Ampicillin/URA3	This study
YIplac211-psy3-K199A,R200A,R201A	Yeast integration	YIplac211	Ampicillin/URA3	This study
pRSF-Duet-CSM2-PSY3	Protein expression	pRSF-Duet	Kanamicin	This study
pRSF-Duet-csm2-K189A,K190A,R191A,R192A-PSY3	Protein expression	pRSF-Duet	Kanamicin	This study
pRSF-Duet-CSM2-psy3-K199A,R200A,R201A	Protein expression	pRSF-Duet	Kanamicin	This study
pRSF-Duet-csm2-K189A,K190A,R191A,R192A-psy3-K199A,R200A,R201A	Protein expression	pRSF-Duet	Kanamicin	This study
pET-DUET-S1S2	Protein expression	pET-DUET	Ampicillin	[8]

Unless noted, all W303 strains are RAD5+ and isogenic to W9100-17D[10] which is derived from W1588[11]
All CG379 strains are isogenic to ySR_128
* Strains derivative from DGY63::171[4]

Supplementary Table 4: PCR oligos

Name	Description	Sequence
oTM-550	*CSM2* deletion cassette generation (forward)	AATAAAAAAAAAATGGAGAGAAGAGACTGCTAGCGGCAAAGGATGCAGCTGAAGCTTCGTACGC
oTM-551	*CSM2* deletion cassette generation (reverse)	GGTGTTACATGGTGTACCGATGCTTTAATTGCACTTATGTAGTCAGCATAGGCCACTAGTGGATCTG
oTM-556	*PSY3* deletion cassette generation (forward)	AAATTCTTAGGAAAAGAGAAAGGAAGTAGCGAATGGAATGGGATGCAGCTGAAGCTTCGTACGC
oTM-557	*PSY3* deletion cassette generation (reverse)	ATTTATGTATCTGAGTTTTTAATGTTTTTTTTCCTTCTCTTATCAGCATAGGCCACTAGTGGATCTG
oTM-546	confirmation of *csm2*Δ::HygMX (forward)	ATTACAAAGAACTCAACTCACTGGC
oTM-549	confirmation of *csm2*Δ::HygMX (reverse)	AATTATTATTACACAGCAGCCCAAG
oTM-552	confirmation of *psy3*Δ::HygMX (forward)	AATCTTCTATTTGGTTGGGTTCTTC
oTM-555	confirmation of *psy3*Δ::HygMX (reverse)	AACTCCACCTTAATACAATTGGACA
oTM-074	Amplification of LEU2 from pUG73 (forward)	CCGCAGGCTAACCGGAACCTGTATT
oTM-075	Amplification of LEU2 from pUG73 (reverse)	GAGCTCGCTGTGAAGATCCCAGCAAAG
oTM-080	Amplification of pySR419 and pySR440 plasmid backbones (forward)	GTTTCTTAGACGTCAGGTGGCACTTT
oTM-081	Amplification of pySR419 and pySR440 plasmid backbones (reverse)	GCCAGAAAATGTTGGTGATGCGC
oTM-092	Amplification of *CAN1* for creation	TATGAGGGTGAGAATGCGAAATGGCG

	of mutation spectra (forward)	
oTM-093	Amplification of *CAN1* for creation of mutation spectra (reverse)	AAGAGTGGTTGCGAACAGAGTAAACC
oTM-094	Sequencing of *CAN1* for creation of mutation spectra	TTGCCACATATCTTCAACGCTGTT
oTM-095	Sequencing of *CAN1* for creation of mutation spectra	AAACTTTGTCACCACCAGTAGATGT
seqDG-91	Sequencing of *CAN1* for creation of mutation spectra	TTTGACAGGGAACAAGTT
Psy3.K199A.Forward	Psy3 site directed mutagenesis at DNA binding residues (forward)	GATAAGTGGTCAATCGCGAGGAAAAGC GGCG
Psy3.K199A.Reverse	Psy3 site directed mutagenesis at DNA binding residues (reverse)	CGCCGCTTTTCCTCGCGATTGACCACT TATC
Psy3.K199A.Add.R2 00A.Forward	Psy3 site directed mutagenesis at DNA binding residues (forward)	GATAAGTGGTCAATCGCGGCGAAAAGC GGCGTTACAC
Psy3.K199A.Add.R2 00A.Reverse	Psy3 site directed mutagenesis at DNA binding residues (reverse)	GTGTAACGCCGCTTTTCGCCGCGATTG ACCACTTATC
Psy3.K199A.R200A. Add.R201A.Forward	Psy3 site directed mutagenesis at DNA binding residues (forward)	GATAAGTGGTCAATCGCGGCGGCAAG CGGCGTTACACTGTACC
Psy3.K199A.R200A. Add.R201A.Reverse	Psy3 site directed mutagenesis at DNA binding residues (reverse)	GGTACAGTGTAACGCCGCTTGCCGCC GCGATTGACCACTTATC
Csm2.K189A.Forwar d	Csm2 site directed mutagenesis at DNA binding residues (forward)	GAACGTCATCTGTGCGTAGCGCAAGAA GGCGGATTAAAAATG
Csm2.K189A.Revers e	Csm2 site directed mutagenesis at DNA binding residues (reverse)	CATTTTTAATCCGCCTTCTTGCGCTACG CACAGATGACGTTC

Csm2.K189A.Add.R190A.Forward	Csm2 site directed mutagenesis at DNA binding residues (forward)	CATCTGTGCGTAGCGCAGCAAGGCGGATTAAAAATG
Csm2.K189A.Add.R190A.Reverse	Csm2 site directed mutagenesis at DNA binding residues (reverse)	CATTTTTAATCCGCCTTGCTGCGCTACGCACAGATG
Csm2.K189A.R190A.Add.R191A.Forward	Csm2 site directed mutagenesis at DNA binding residues (forward)	GTGCGTAGCGCAGCAGCGCGGATTAAAAATG
Csm2.K189A.R190A.Add.R191A.Reverse	Csm2 site directed mutagenesis at DNA binding residues (reverse)	CATTTTTAATCCGCGCTGCTGCGCTACGCAC
Csm2.K189A.R190A.R191A.ADDR191A.Forward	Csm2 site directed mutagenesis at DNA binding residues (forward)	CGTAGCGCAGCAGCGGCGATTAAAAATGGAG
Csm2.K189A.R190A.R191A.ADDR191A.Reverse	Csm2 site directed mutagenesis at DNA binding residues (reverse)	CTCCATTTTTAATCGCCGCTGCTGCGCTACG
Csm2.S2	Csm2 6xHA tagging cassette generation (forward)	GTACTGGTGTTACATGGTGTACCGATGCTTTAATTGCACTTATGTAGTCAATCGATGAATTCGAGCTCG
Csm2.S3	Csm2 6xHA tagging cassette generation (reverse)	ATTCCCTTGCTGAATATATCTGGAAGTATTATGCAGATTCATTATTCGAACGTACGCTGCAGGTCGAC
Csm2.CKF2	Csm2 6xHA tagging confirmation (forward)	AATGGAGAGAAGAGACTGCTAGCG
Csm2.CKR2	Csm2 6xHA tagging confirmation (reverse)	AGTCTAGCATCGGGGTAGTTTTCC
Psy3.S2	Psy3 6xHA tagging cassette generation (forward)	ATTTAATTTATGTATCTGAGTTTTTAATGTTTTTTTTCCTTCTCTTATCAATCGATGAATTCGAGCTCG
Psy3.S3	Psy3 6xHA tagging cassette generation (reverse)	AAGTTGTTGACGGCAGGCCACAGTACAGAAGGATAGCCGCACTTGAAGAACGTACGCTGCAGGTCGAC
Psy3.CKF2	Psy3 6xHA tagging	TGTGTACCGTAAGCATTACTCC

	confirmation (forward)	
Psy3.CKR2	Psy3 6xHA tagging confirmation (reverse)	TCCTGGTAGATGTAAGCATTGC
KanHisNat	6xHA tagging confirmation sequencing	GACTGTCAAGGAGGGTATTCTG

Supplementary Table 5: Sequence of abasic site analog containing DNA

Name	Sequence	Mass Calculated	Mass Found
AP1	TTT TTT TTT XTT TTT TTT TTC TTG ACA AGC TTG CGC ACT G	12,053.8	12,054.4 [M+H]
AP2	TTT TTT TTT TTT TTT TTT TXC TTG ACA AGC TTG CGC ACT G	12,053.8	12,052.4 [M-H]
AP3	TTT TTT TTT TTT TTT TTT TTX TTG ACA AGC TTG CGC ACT G	12,052.8	12,049.5 [M-3H]
AP4	TTT TTT TTT TTT TTT TTT TTC TTG ACA AGC XTG CGC ACT G	12,053.8	12,054.9 [M+H]
AP5	CAG TGC CGA AGC TTG TCA AGT TTT TTT TTT XTT TTT TTT T	12,101.9	12,099.7 [M-2H]
AP6	CAG TGC CGA AGC TTG TCA AGX TTT TTT TTT TTT TTT TTT T	12,101.9	12,099.7 [M-2H]
AP7	CAG TGC CGA AGC TTG TCA AXT TTT TTT TTT TTT TTT TTT T	12,076.8	12,077.8 [M+H]
AP8	CAG TGC CGA XGC TTG TCA AGT TTT TTT TTT TTT TTT TTT T	12,092.9	12,092.4 [M]

Supplementary Table 6: Oligos for additional DNA fork substrates

Name	Sequence
X01	GACGCTGCCGAATTCTACCAGTGCCTTGCTAGGACATCTTTGCCCACC TGCAGGTTCACCC-Cy3
X04*	ATCGATAGTCGGATCCTCTAGACAGCTCCAT(dU)TAGCAAGGCACTGGT AGAATTCGGCAGCGT
DS2	TGGGTGAACCTGCAGGTGGGCAAAGATGTCC
DS3*	ATGGAGCTGTCTAGAGGATCCGACTATCGA

* Denotes single nucleotide change in substrate. As originally published in [1].

Supplementary References

1 Zheng, X. F. *et al.* Processing of DNA structures via DNA unwinding and branch migration by the *S. cerevisiae* Mph1 protein. *DNA Repair (Amst)* **10**, 1034-1043 (2011).

2 Godin, S. K. *et al.* Evolutionary and functional analysis of the invariant SWIM domain in the conserved Shu2/SWS1 protein family from Saccharomyces cerevisiae to Homo sapiens. *Genetics* **199**, 1023-1033 (2015).

3 Godin, S. K. *et al.* The Shu complex promotes error-free tolerance of alkylation-induced base excision repair products. *Nucleic Acids Research* **44**, 8199-8215 (2016).

4 James, P., Halladay, J. & Craig, E. A. Genomic libraries and a host strain designed for highly efficient two-hybrid selection in yeast. *Genetics* **144**, 1425-1436 (1996).

5 Roberts, S. A. *et al.* Clustered mutations in yeast and in human cancers can arise from damaged long single-strand DNA regions. *Molecular Cell* **46**, 424-435 (2012).

6 Hoopes, J. I. *et al.* APOBEC3A and APOBEC3B Preferentially Deaminate the Lagging Strand Template during DNA Replication. *Cell Reports* **14**, 1273-1282 (2016).

7 Goldstein, A. L. & McCusker, J. H. Three new dominant drug resistance cassettes for gene disruption in *Saccharomyces cerevisiae*. *Yeast* **15**, 1541-1553 (1999).

8 Gaines, W. A. *et al.* Promotion of presynaptic filament assembly by the ensemble of *S. cerevisiae* Rad51 paralogues with Rad52. *Nature Communications* **6**, 7834, (2015).

9 Knop, M. *et al.* Epitope tagging of yeast genes using a PCR-based strategy: more tags and improved practical routines. *Yeast* **15**, 963-972 (1999).

10 Thomas, B. J. & Rothstein, R. Elevated recombination rates in transcriptionally active DNA. *Cell* **56**, 619-630 (1989).

11 Zhao, X., Muller, E. G. & Rothstein, R. A suppressor of two essential checkpoint genes identifies a novel protein that negatively affects dNTP pools. *Molecular Cell* **2**, 329-340 (1998).
